# Imported cases of malaria in Spain: observational study using nationally reported statistics and surveillance data, 2002–2015

**DOI:** 10.1186/s12936-019-2863-2

**Published:** 2019-07-10

**Authors:** Zaida Herrador, Beatriz Fernández-Martinez, Víctor Quesada-Cubo, Oliva Diaz-Garcia, Rosa Cano, Agustín Benito, Diana Gómez-Barroso

**Affiliations:** 1 National Centre for Tropical Medicine, Health Institute Carlos III (ISCIII), 28029 Madrid, Spain; 2 Network Biomedical Research on Tropical Diseases (RICET in Spanish), Madrid, Spain; 3 National Centre for Epidemiology, Instituto de Salud Carlos III (ISCIII), Madrid, Spain; 4 Consortium for Biomedical Research in Epidemiology and Public Health (CIBERESP), Madrid, Spain; 5 Hospital Universitario Gregorio Marañón, Madrid, Spain

**Keywords:** Malaria, Imported malaria, Malaria chemoprophylaxis, Non-endemic areas, Spain

## Abstract

**Background:**

Malaria was eliminated in Spain in 1964. Since then, more than 10,000 cases of malaria have been reported, mostly in travellers and migrants, making it the most frequently imported disease into this country. In order to improve knowledge on imported malaria cases characteristics, the two main malaria data sources were assessed: the national surveillance system and the hospital discharge database (CMBD).

**Methods:**

Observational study using prospectively gathered surveillance data and CMBD records between 2002 and 2015. The average number of hospitalizations per year was calculated to assess temporal patterns. Socio-demographic, clinical and travel background information were analysed. Bivariate and multivariable statistical methods were employed to evaluate hospitalization risk, fatal outcome, continent of infection and chemoprophylaxis failure and their association with different factors.

**Results:**

A total of 9513 malaria hospital discharges and 7421 reported malaria cases were identified. The number of reported cases was below the number of hospitalizations during the whole study period, with a steady increase trend in both databases since 2008. Males aged 25–44 were the most represented in both data sources. Most frequent related co-diagnoses were anaemia (20.2%) and thrombocytopaenia (15.4%). The risks of fatal outcome increased with age and were associated with the parasite species (*Plasmodium falciparum*). The main place of infection was Africa (88.9%), particularly Equatorial Guinea (33.2%). Most reported cases were visiting friends and relatives (VFRs) and immigrants (70.2%). A significant increased likelihood of hospitalization was observed for children under 10 years (aOR:2.7; 95% CI 1.9–3.9), those infected by *Plasmodium vivax* (4.3; 95% CI 2.1–8.7) and travellers VFRs (1.4; 95% CI 1.1–1.7). Only 4% of cases reported a correct regime of chemoprophylaxis. Being male, over 15 years, VFRs, migrant and born in an endemic country were associated to increased risk of failure in preventive chemotherapy.

**Conclusions:**

The joint analysis of two data sources allowed for better characterization of imported malaria profile in Spain. Despite the availability of highly effective preventive measures, the preventable burden from malaria is high in Spain. Pre-travel advice and appropriately delivered preventive messages needs to be improved, particularly in migrants and VFRs.

## Background

According to the World Health Organization (WHO), between 2000 and 2015 there has been a 37% global decrease in malaria incidence and a 60% decrease in global mortality rates [1]. Meantime, increasing travel to endemic areas in recent decades in association with the significant influx of immigrants from malaria-endemic countries has brought a steady increase in the number of imported cases in non-endemic countries [2].

Malaria poses a serious health hazard for travellers to areas of endemicity. Imported malaria refers to infections acquired outside and brought into a national territory [3]. Imported cases to non-endemic countries often result in delays in diagnosis, are expensive to treat, and can sometimes cause secondary local transmission [4]. In 2016, the European Region was the first in the world to have achieved interruption of indigenous malaria transmission. Still, malaria is the imported disease with the highest number of notifications in Europe [5]. It has been estimated that every year 10–15 million international travellers from Europe visit malaria endemic areas and 12,000–15,000 cases of malaria are imported into the EU, with an average fatality rate of 0.4–3% [6]. The European Centre for Disease Prevention and Control (ECDC) coordinates this disease surveillance in the European Union (EU) and the member countries of the European Free Trade Association (EFTA). In 2015, the largest number of confirmed cases in this region was notified by France, followed by the UK and Spain (2500, 1397 and 706 cases, respectively) [7].

Spain was declared free of malaria in 1964. In the last decades, notified malaria infections have been mostly imported [8, 9]. Cases by autochthonous transmission have been scarce, and mainly related to health care (transfusion, transplants, parenteral or nosocomial), or vertical transmission. The cases of airport malaria have been anecdotal, and only 2 recent cases of malaria introduced by *Plasmodium vivax* have been documented [10]. Although there is a wide distribution of the potential vector of this species, it is considered that the current risk of introduced malaria is low [8]. Confirmed cases are monitored through the National Network of Epidemiological Surveillance (RENAVE in Spanish, Royal Decree 2210/1995) [11]. The last update of the malaria surveillance protocol was carried out in 2013 [12]. Other alternative source of information is the Centralized Hospital Discharge Database (CMBD in Spanish).

Reports have shown that knowledge on infectious disease prevention among departing travellers and the adherence of travellers to WHO and Centers for Disease Control and Prevention (CDC) recommendations is far from optimal [13]. In Spain, pre-travel consultation is voluntary (except for the yellow fever vaccination, which is mandatory at the entrance of several countries). Preventive measures and chemoprophylaxis against malaria follow the WHO recommendations, and depend on the travel destination, the duration of potential exposure, parasite resistance pattern, level and seasonality of transmission, age and pregnancy [14]. In order to improve knowledge on malaria imported cases characteristics, the epidemiological and clinical characteristics of patients diagnosed with malaria within the CMBD and the RENAVE databases were assessed. The hospitalization risk factors and the possible association between malaria chemoprophylaxis intake and gender, age or travel reason, among other factors, were also explored.

## Methods

### Data source

An epidemiological study using the CMBD and the RENAVE database for the time period January 1st, 2002 to December 31st, 2015 was performed.

### CMBD

The CMBD database receives notification from around 98% of the public hospitals in Spain [15]. The National Health System (NHS) provides free medical care to 99.5% of the Spanish population, although those persons not covered by the NHS can be attended at the public hospitals. Private hospitals represent only a small proportion of all hospital admissions. Since 2005, CMBD also has a gradual coverage from private hospitals [16].

International Classification of Diseases, Ninth Revision, Clinical Modification (ICD-9CM), the ICD version employed during the study period, was used for this purpose [17]. Registers with ICD-9 CM “malaria” and “malaria complicating pregnancy childbirth or the puerperium” codes (“084.*”; “647.4”) placed in any diagnostic position were analysed. The database was cleaned to remove any potential duplicates of hospitalizations. Sociodemographic and clinical data were collected. Relevant malaria related co-diagnoses were also explored.

### RENAVE

Surveillance on malaria is comprehensive in Spain and based on aggregated and case-based notification. Case definition includes probable (patient who meet clinical criteria and with history of travel or permanence in an endemic area) and confirmed (+ laboratory confirmation by thick blood smear, detection of *Plasmodium* nucleic acid and/or a positive rapid diagnostic test). Regional public health authorities (autonomous regions) should report aggregated cases weekly and complete the reporting form information as soon as possible, using a standardized questionnaire [12]. Consistent individualized malaria data is available from 2002 on (and it is exhaustive at national level since 2014). Non-imported cases, which are notified urgently, were excluded for the analysis.

For each entry, socio-demographic, clinical and travel background information were analysed. Age was categorized in five groups: 0–15, 16–24, 25–34, 35–44 and ≥ 45 years old. Place of birth and travel information were only available from RENAVE and includes place of birth and travel (country or continent when country is unknown), date and reason for travel (tourism, visiting friends and relatives (VFRs), work or being immigrant—this category includes people who had spent more than 1 year living in an endemic area, independently of their nationality).

### Statistical analysis

The average number of hospitalizations per year was calculated in order to assess temporal patterns. Population figures of the Spanish municipalities were obtained from the Spanish National Statistics Institute [18] and were used as denominators for the study period, for both CMBD and RENAVE data.

Frequencies and percentages were used to summarize CMBD and RENAVE data. Differences in proportions were assessed by the χ^2^ test and 95% confidence intervals (95% CI) were calculated. ANOVA was used to compare differences in means. Two-sided tests were used and p < 0.05 was considered significant.

Bivariate analyses for continent of infection, and relevant related factors were performed for RENAVE data. Bivariate analyses preceding logistic regression models for preventive chemotherapy (RENAVE), fatal outcome (CMBD and RENAVE) and hospitalization (RENAVE) were also carried out. Probable cases were excluded from the multivariate regression analysis, which were obtained by using a manual backward stepwise procedure. Age and gender, considered biologically relevant, and all variables associated with each of the outcomes at the p < 0.10 level were included in the multivariable analysis. The major assumptions of logistic regression analysis (absence of multicollinearity and interaction among independent variables) were checked to be satisfied. The goodness of fit was assessed using Hosmer–Lemeshow statistic. The adjusted odds ratio (aOR) and 95% CI were computed. p-values less than or equal to 0.05 were considered statistically significant. Data analysis was performed using STATA software version 14.

### Ethics statement

This study involves the use of patient data from The Spanish Centralized Hospital Discharge Database (CMBD) and the RENAVE. CMBD data are hosted by the Ministry of Health, Consumption and Social Welfare (MSCBS in Spanish). Researchers working in public and private institutions can request the databases by filling, signing and sending a questionnaire available at the MSCBS website. A signed Confidentiality Commitment is required in this questionnaire. All data are anonymized and de-identified by the MSCBS before it is provided to applicants. According to this Confidentiality Commitment signed with the MSCBS, researchers cannot provide the data to other researchers that must request the data directly to the MSCBS [15]. RENAVE data are registered through the national reporting electronic platform (SiViEs in Spanish) and hosted by the National Centre of Epidemiology. The so-called “SiViEs” computer platform was designed for epidemiological surveillance in Spain. It meets all legal and technical requirements concerning safe access and data protection. Formal ethical approval is not required for routine surveillance activities in Spain.

## Results

**Fig. 1:**
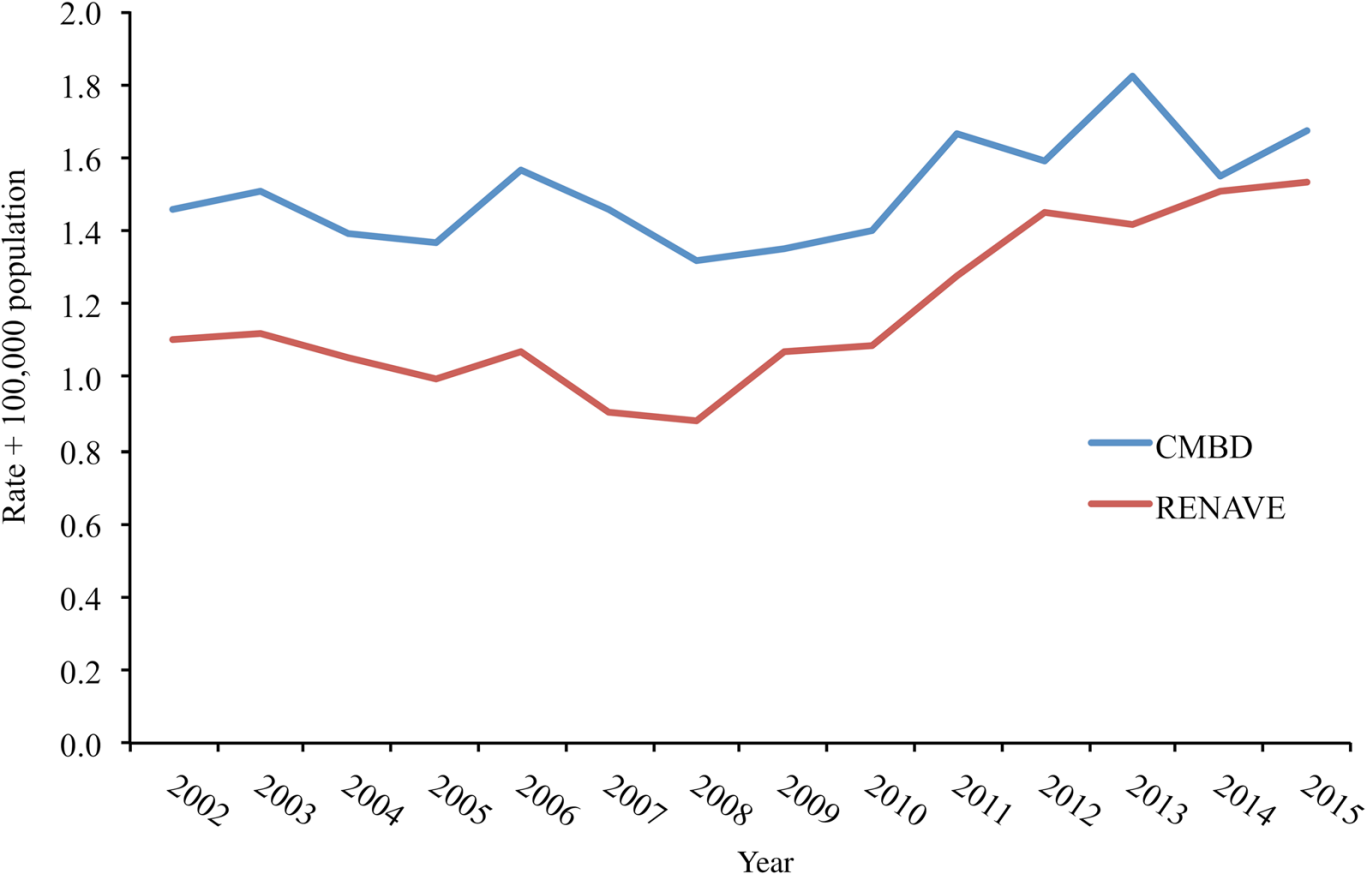
Imported malaria incidence rates per 100,000 population based on CMBD and RENAVE records, Spain, 2002–2015. RENAVE data is based in individual case reports, except for one region. In this case, the number of cases per year were replaced by aggregated data. Starting from 2014, RENAVE individualized data are comprehensive for the whole country

A total of 9513 hospital discharges related to malaria (ICD-9-CM codes 123.*) and 7421 reported cases of malaria (6060 imported cases with individualized data) were identified for the 14-year study period. The number of reported cases was below the number of hospitalizations during the whole study period, although the difference between both records decreased over time (Fig. 1).

**Fig. 2:**
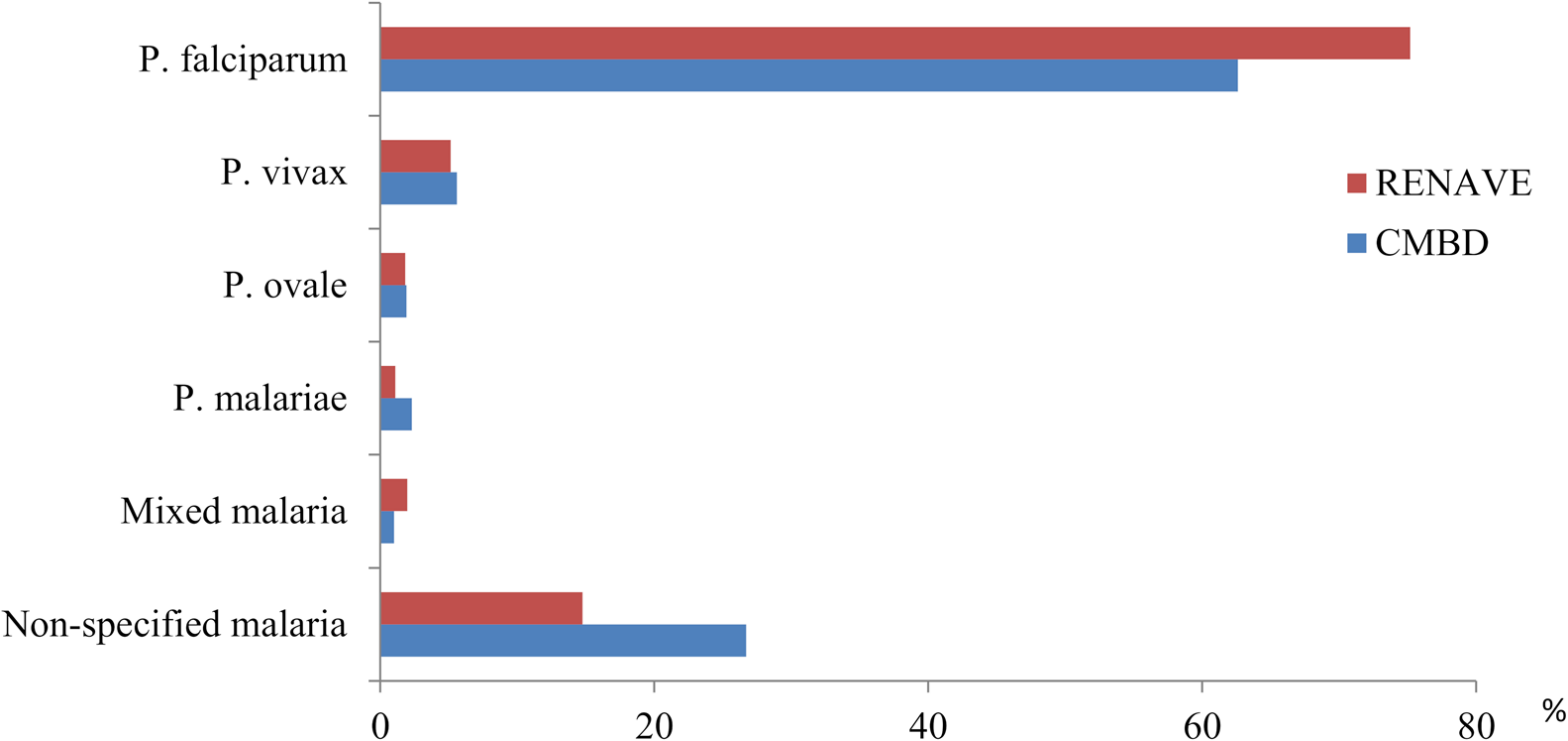
Type of isolated Plasmodium, CMBD and RENAVE, Spain, 2002–2015

The most frequent isolated species of *Plasmodium* in both databases was *Plasmodium falciparum.* 26.7% and 14.7% cases were non-specified malaria in CMBD and RENAVE, respectively (p < 0.01) (Fig. 2).

According to CMBD records, the mean age of the 9513 hospitalized patients was 31.9 years [interquartile range (IQ) = 23–41], being the 25–34 and 35–44 age groups the most represented. A total of 63.8% hospitalized were males, particularly predominating above 25 years old groups. There were 262 hospitalized women with pregnancy-associated malaria.

The majority (94.6%) of malaria patients with known source of admission were admitted from emergency rooms. 95.8% of hospitalizations were discharged at home, death occurring in 0.8%. The risk of fatal outcome increased with age (mean of 50.4 vs. 31.7 years old in exitus and non-exitus, respectively; aOR: 1.05; 95% CI 1.03–1.07). The exitus outcome was also more frequent among those hospitalized with mixed malaria (aOR: 4.21; 95% CI 1.45–12.21). The hospitalization median time was 4 days (IQ range = 3–6), with a median cost of 3995 € (Table 1).

**Table 1:** Sociodemographic and clinical characteristics of malaria related hospitalizations, CMBD 2002–2015, Spain

Variable	n (%)
Gender	
Male	6064 (63.8)
Female	3442 (36.2)
Age-groups (years)	
< 15	1643 (17.3)
15–24	991 (10.4)
25–34	2739 (28.8)
35–44	2329 (24.5)
≥ 45	1810 19)
Type of admission	
Urgent	8998 (94.6)
Programmed	507 (5.3)
Others/unknown	8 (0.1)
Type of discharge	
Home	9113 (95.8)
Transfer	186 (2)
Voluntary discharge	110 (1.2)
Others/unknown	24 (0.3)
Exitus	80 (0.8)
Hospitalization time	
< 1 week	7154 (75.2)
≥ 1 week	2359 (24.8)

	Mean	Median (IQ range)
Age (years)	31.9	33 (23–41)
Hospitalization time (days)	5.9	4 (3–6)
Hospitalization cost (euro)	4149.3	3994.9 (3062–4296)

Most frequent related co-diagnoses were anaemia (20.2%, mostly iron deficiency anaemias and acquired and hereditary haemolytic anaemias), thrombocytopaenia (15.4%; ICD-9-CM codes: 287.3-5), splenomegaly (2.4%; ICD-9-CM code: 789.2) and hepatomegaly (1.5%; ICD-9-CM code: 789.3). Other frequent co-diagnoses were HIV (4.8%; ICD-9-CM codes: 042, V08), acute kidney failure/non-specified (4.2%; ICD-9-CM code: 584, 586) and helminthiases (3.1%; ICD-9-CM code: 120–129).

In the RENAVE, epidemiological questionnaire were available for 6060 out of 7421 (81.7%) reported cases of malaria. The 64.7% were males. The mean age was 32.6 (IQ range = 25–41). More than 50% were between 25 and 45 years of age. The majority were born in a country other than Spain (66.2%), being Africa the most frequent continent of birth (59.0%). The main place of infection was Africa (88.9%), particularly Equatorial Guinea (33.2%). Most reported cases were VFRs and immigrants (70.2%). Only 4% referred a correct regime of chemoprophylaxis (Table 2).

**Table 2:** Characteristics of imported malaria cases (RENAVE), Spain, 2002–2015

Variable	n (%)
Gender	
Male	3923 (64.7)
Female	2106 (34.8)
Unknown	31 (0.5)
Age-groups (years)	
< 15	865 (14.3)
15–24	596 (9.8)
25–34	1791 (29.6)
35–44	1612 (26.6)
≥ 45	1148 (18.9)
Unknown	48 (0.8)
Classification	
Confirmed	5850 (96.5)
Probable	210 (3.5)
Hospitalization	
No	815 (13.5)
Yes	4543 (74.8)
Unknown	702 (11.6)
Exitus	
No	3872 (63.9)
Yes	35 (0.6)
Unknown	2153 (35.5)
Country of residence	
Spain	3972 (65.6)
Other	365 (6.0)
Unknown	1723 (28.4)
Place of birth	
Spain	1216 (20.1)
Other	4010 (66.2)
Unknown	834 (13.8)
Continent of birth	
Africa^a^	3576 (59.0)
America	150 (2.5)
Asia	121 (2.0)
Oceania	5 (0.1)
Europe	1341 (22.1)
Unknown	867 (14.3)
Travel reason	
VFRs	2770 (45.7)
Immigrant	1482 (24.5)
Tourism	610 (10.1)
Work	695 (11.5)
Unknown	503 (8.3)
Place of infection	
Africa^b^	5389 (88.9)
America	201 (3.3)
Asia	154 (2.5)
Oceania	14 (0.2)
Unknown	302 (5.0)
Chemoprophylaxis	
None	3020 (49.8)
Non complete	626 (10.3)
Complete	243 (4.0)
Unknown	2171 (35.8)

*VFRs* visiting friends and relatives

^a^1349 (37.7%) from Equatorial Guinea

^b^2009 (37.3%) from Equatorial Guinea

74.8% of reported cases had required hospitalization. A significant increased likelihood of hospitalization was observed for children under 10 years (aOR: 2.7; 95% CI 1.9–3.9), those infected by *P. vivax* (4.3; 95% CI 2.1–8.7), mixed *Plasmodium* infections (aOR: 3.2; 95% CI 1.4–7.5), *P. falciparum* (aOR: 3.1; 95% CI 1.7–5.6), and *Plasmodium ovale* (aOR= 2.3; 95% CI 1.1–5.2), being *Plasmodium malariae* the reference category, and VFRs (1.4; 95% CI 1.1–1.7) with respect to other travellers.

Fatal outcome was reported for 35 cases (0.6%), from whom 30 were due to *P. falciparum* and 1 to mixed *P. falciparum* and *P. ovale* malaria, all infected in Africa (unknown species in 4 cases). The risk of fatal outcome increased with age (aOR: 1.04; 95% CI 1.02–1.07) and for those born in Europe (5.4; 95% CI 1.5–20.2), independently of the reason for travel.

In 95% of the records, the most likely continent of infection was supplied (Table 2). Gender distribution was similar for all the continents. Cases coming from Asia were significantly younger than those infected in other continents (p < 0.01). More than a half of patients infected in Africa were VFRs, while there was a higher proportion of tourists among cases coming from America and Asia (p < 0.01). Infections acquired in Asia and America were, for the greater part, caused by *P. vivax* (78.5% and 61.1%, respectively), whereas those acquired in Africa were mainly caused by *P. falciparum* (92.3%) (Table 3).

**Table 3:** Characteristics of reported cases by continent of infection, RENAVE, Spain 2002–2015

Variables	Africa (n = 5389)		America (n = 201)		Asia (n = 154)		Oceania (n = 14)		p value
	n	%	n	%	n	%	n	%	
Gender									
Male	3502	65.3	122	61.0	103	66.9	9	64.3	NS
Female	1860	34.7	78	39.0	51	33.1	5	35.7	
Age group (years)									
< 15	781	14.6	9	4.5	31	20.4	0	0.0	< 0.001
15–24	504	9.4	32	16	28	18.4	1	7.1	
25–34	1566	29.2	82	41	46	30.3	3	21.4	
35–44	1475	27.5	42	21	23	15.1	7	50.0	
≥ 45	1030	19.2	35	17.5	24	15.8	3	21.4	
Continent of birth									
Africa	3571	74.1	0	0.0	2	1.4	0	0.0	< 0.001
America	43	0.9	105	57.1	0	0.0	1	10.0	
Asia	27	0.6	1	0.5	88	62.9	0	0.0	
Oceania	3	0.1	0	0.0	0	0.0	2	20.0	
Europe	1178	24.4	78	42.4	50	35.7	7	70.0	
Reason for travel or stay									
Immigrant	1360	26.4	64	32.8	42	30.2	3	25.0	< 0.001
Work	654	12.7	19	9.7	10	7.2	3	25.0	
Tourism	494	9.6	67	34.4	34	24.5	5	41.7	
VFRs	2642	51.3	45	23.1	53	38.1	1	8.3	
Chemoprophylaxis									
None	3790	77.9	113	83.9	95	89.0	2	37.5	< 0.001
Non complete	589	15.5	18	10.2	11	7.6	3	25.0	
Complete	226	6.6	7	5.9	4	3.4	3	37.5	
*Plasmodium species*									
*P. falciparum*	4374	92.3	55	34.0	20	14.8	5	45.5	< 0.001
*P. vivax*	92	1.9	99	61.1	106	78.5	5	45.5	
*P. ovale*	102	2.2	0	0.0	5	3.7	1	9.1	
*P. malariae*	57	1.2	3	1.9	3	2.2	0	0.0	
Mixed	112	2.4	5	3.1	1	0.7	0	0.0	
Hospitalization									
No	736	15.0	28	15.5	12	8.3	0	0.0	NS (p = 0.076)
Yes	4180	85.0	153	84.5	132	91.7	11	100.0	

Being male and older than 15 years old were associated to increased risk of failure in preventive chemotherapy (considering failure as none or non-complete preventive treatment) (Table 3). VFRs were 1.8 times more prompted to fail in malaria preventive chemotherapy than those travelling for work purposes. The risk was even higher for immigrants (aOR: 3.3; 95% CI 2.2–4.9). Malaria cases born in endemic countries were at higher risk of failure in preventive chemotherapy (Table 4).

**Table 4:** Risk factors for failure in preventive chemotherapy, RENAVE, Spain 2002–2015

Variables	aOR	95% CI	p value
Age group (ref.: < 15 years) (years)			
15–24	1.1	1.1–2.4	0.009
25–34	1.6	1.2–2.3	0.001
35–44	1.8	1.3–2.4	< 0.001
45+	1.5	1.1–2.1	0.011
Gender (ref.: female)			
Male	1.3	1.1–1.6	0.002
Reason for travel or stay (ref.: work)			
Immigrant	3.3	2.2–4.9	< 0.001
Tourism	1.4	1.0–1.9	0.040
VFR	1.8	1.3–2.5	< 0.001
Born in malaria endemic country (ref.: no)^a^			
Yes	2.3	1.7–3.0	< 0.001

^a^Countries endemic for malaria by 2015 [1]

## Discussion

Overall, a slight increase in the incidence rates of imported malaria during the study period was found in both databases. According to the WHO, there have been important gains in malaria control over the past two decades worldwide, although this progress has stalled in many countries in the most recent years [19]. In the European Region, 45 countries reported a decline in imported malaria cases and deaths between 2001 and 2010, possibly reflecting malaria control activities in endemic countries, an increase in the number of countries classified as malaria-free and/or and a possible under-reporting of cases [3]. Later on, at EU level the trend was upward until 2011, reduced in 2012 and again increased since 2013 [20]. Most probably, the difference in imported malaria trends in Spain with other European countries is explained by differences in the architecture of the air network, historical ties (in fact, the 22.3% of malaria reported cases in Spain were born in Equatorial Guinea, a former Spanish colony, and highly endemic to malaria [21]), sociodemographic characteristics of travellers, and malaria endemicity, among other factors [13, 19]. On the other hand, this increase coincides with an increase in immigration figures in Spain, although this increase was halted in 2010 mainly due to the economic crisis [22], which would not explain the increase in reported cases and hospitalizations in the last years of study. Nevertheless, it should be taken into account that African immigrants’ figures remained more or less constant during the economic crisis [22, 23].

The comparison of compulsory notified disease records with hospital records indicates a discrepancy between both registries, as already stated by other authors for other infectious diseases [24, 25]. Moreover, if it is assumed that not all malaria cases require hospitalization (74.8% according to RENAVE figures), then the total number of malaria cases occurring in Spain is even higher. Difficulties in reporting of imported malaria do not apply to Spain alone. Comparison of notified cases with hospital records have indicated a clear discrepancy in several countries [26, 27]. Nevertheless, this comparison should be read with caution, as the number of hospitalized malaria cases might be overrated due to re-admissions and/or misclassification.

Among all malaria cases, the most frequent diagnosis was *P. falciparum.* The high prevalence of *P. falciparum* is in accordance with its well-documented relative virulence, the global prevalence of this species and also with other reports of imported malaria, mainly in patients returning from sub-Saharan Africa [1, 2, 28]. In the UK, this species accounted for about 70% of cases notified in 2011, whereas 25% of cases were due to *P. vivax* [29, 30]. In Spain, the % of imported cases due to *P. vivax* was quite lower, probably due to differences in travel destinations and immigrants’ country of origin. On the other hand, a worthy of consideration number of reported cases and related hospitalizations miss microbiological information. An improvement in using complementary information from laboratory based surveillance system may resolve this problem as well as improve the surveillance performance.

In both databases, the distribution by gender and age group was similar. The proportion of malaria-related hospitalizations and case reporting was higher for males than females. Major risk of malaria among male travellers has been well documented [31]. Compared with women, men seem to be less likely to seek pre-travel advice, to adhere to appropriate personal vector avoidance and chemoprophylaxis, suffer more mosquito bites, and exhibit other high-risk behaviours [27, 32]. In fact, in this study the risk of failure in preventive chemotherapy was higher in males, result that support these hypotheses. The age distribution of malaria cases, which may reflect the age distribution of international travellers and immigrants or expatriates from endemic areas, is also consistent with previous reports [6, 28].

The median hospitalization stay was below 5 days, and the rate of in hospital deaths and case-fatality was under 1%. Available treatment regimens for malaria in most non-endemic countries are highly effective when properly and promptly administered, and symptoms can resolve within days [33]. The risk of hospitalization was higher for children under 10 years, while case fatality increased with age. Young children are at a higher risk of acquiring malaria abroad, while complicated disease occur more frequently at older ages [34]*. P. vivax* infection resulted in an increased risk of hospitalization*. P. vivax* infection, common in tropical countries in the Americas, Central and Southeast Asia, and Oceania [35], usually leads to milder disease and relapses. However, in recent years, many cases of severe malaria have been reported in *P. vivax* and *P. knowlesi* malaria. This seems to be related to travellers’ non-immune status, no antimalarial prophylaxis intake (as travellers are less aware of malaria risk in these areas), delay in treatment, and severity of the illness at admission of travelers [36]. In fact, in this study the proportion of tourists among travellers returning from *P. vivax* endemic countries was higher than from other continents.

Death risk was higher among cases with mix infections and those infected with *P. falciparum*, which is consistent with the literature [1, 3, 37]. Interestingly, case fatality was higher for those born in Europe (5.4; 95% CI 1.5–20.2), independently of the reason for travel. It is known that malaria partial immunity in VFRs wanes with time resulting, especially after 12 years, in a more serious malaria clinical presentation [6, 38]. Thus, it could have been expected that this group showed the highest fatality rate. Nevertheless, it should be taken into account that the overall fatality rate was fairly low for meaningful analysis.

More than a half reported cases were born in Africa, and travellers, VFRs and immigrants accounted for the majority of imported malaria cases, as documented in several studies [28, 39]. Moreover, VFRs were less likely to report the use of any chemoprophylaxis. VFRs and immigrants from endemic countries are high risk groups for malaria due to their behavioral patterns and for geographical reason. The risk of infection vary, and is a function of several factors, including: the transmission intensity of the origin location; the activities undertaken in their visits; and prophylaxis availability and adherence [4, 40]. Also importantly, these individuals may perceive themselves to be immune or at low risk, and may forego malaria prevention measures [27].

Malaria cases from Asia were significantly younger than those infected in other continents. A possible explanation might be that Asian migrants travel with their family members more frequently than migrants coming from Africa [41]. Moreover, there was a higher % of tourists among cases coming from America and Asia. Although there is no risk of malaria in many tourist destinations in south-east Asia, the Caribbean and Latin America [1], malaria is still prevalent in other Southeast Asian and Latin American areas where large numbers of backpackers visit each year [3]. Furthermore, these travellers’ risk perception might be low and thus affect the pre-travel health-seeking practices [42].

Overall, more than 60% of imported cases referred incomplete or none chemoprophylaxis. This % was particularly high among VFRs and migrants. According to the CDC, failure of prophylaxis may occur for at least three reasons. First, travellers may not seek or follow advice, or may receive inaccurate advice. Second, travellers may forget or not completely understand chemoprophylactic advice, or they may even be advised by peers not to use chemoprophylaxis. Third, general physicians infrequently provide pre-travel advice to patients and may not be aware of the current recommendations [43]. It is agreed that VFRs and immigrants are malaria risk groups requiring special attention [2, 27, 31]. Particularly, VFRs seem to be less inclined than other travellers to get pre-travel advice and to use chemoprophylaxis against malaria [13]. According to Scolari et al. in Italy, around 80% of migrants and VFRs do not have adequate information and do not take preventive measures during travel, although they are aware of the malaria risk in their origin countries [44]. In a recent qualitative study, it was observed that an important determinant that explained preventive behavior was the opinion that curing malaria is easier than using preventive tablets [45]. Moreover, recommendations for malaria chemoprophylaxis may often fail to address the cultural, social and economic needs of VFRs [46]. Finally, several studies in Spain found that migrants use health services differently from natives: they attend more frequently general practitioner (GP) practices and emergency rooms, especially those migrants from low-income countries [47, 48]. This problem has been recently approached in UK, by approving the switch of anti-malarial from a prescription-only medicine to a pharmacy medicine [49]. In Spain, malaria chemoprophylaxis is considered to be as a medical physician competence. This measure may not be appropriate in this country for the following reasons (among others): (a) anti-malarial prescription might be accurately assessed if it is necessary or not, depending on the destination and type of travel. According to the traveller’s profile and his/her medical conditions, the appropriate drug and regimen would be decided. Otherwise, it might not be secure for travellers, creating scope to hasten the problem of antimicrobial immunity; (b) it may pose a challenge for the worldwide increase in antimicrobial resistance, and (c) Spanish pharmacists may not be prepared to give this kind of health advice, i.e. they do not have quick access to the latest information on what antimalarial is suitable to a given geography at a given time. In Spain, more suitable alternatives to improve malaria prevention in travellers may be: to reduce the price of prescribed anti-malarials; to make health services for immigrants and travellers (particularly VFRs) more readily available and adapted to these risk groups; and to improve and expand the pre-travel advice at primary health care level.

## Limitations and conclusions

This study has several limitations. Despite that two official databases (RENAVE and CMBD) were analysed, must probably the real burden of imported malaria in Spain is still underestimated. On one hand, hospital discharge records do not include cases managed in outpatient settings or asymptomatic cases, thus hospital records are still underestimating the real burden of malaria. Moreover, CMBD remains dependent by the population’s health seeking behavior and healthcare accessibility [50]. On the other hand, underreporting of cases by national surveillance systems is common issue in Europe [4, 6]. It is important to address the constraints leading to underreporting, namely providing education and feedback to relevant health care workers on the importance of the notification process. Other limitation is the lack of denominator (overall number of travellers by origin and destination). Nevertheless, CMBD and RENAVE data are representative of the imported cases of Spain. Furthermore, it is the first time that both databases are analysed together in this particular issue.

Both analysed databases miss relevant information, such as personal and travel information, which may be useful to further explore and explain the raised hypotheses. These calls for the need to undertake further (qualitative and quantitative) investigations not only to substantiate these results but also to check new hypothesis which may have emerged.

This study confirms that the risk of imported malaria is higher in travellers from Africa, especially immigrants and VFRs, and that male and patients at the extremes of age constitute groups with increased risks. This study results also points that failure in preventive chemotherapy is still too common among imported malaria cases, particularly among those groups. All pre-travel advice needs to be individualized for each traveller, based on the traveller cultural background, exact travel route, season, and type of travel.

Finally, data on the features of imported cases can also provide valuable information about both the epidemiology of malaria in endemic regions, and on how malaria moves around the world. Moreover, with *Anopheles* vectors still present, imported cases can also cause secondary transmission in Spain, although the chances of resumption of endemic transmission are very small. This is mainly due to the fact that circulating anopheles in Spain are only competent for *P. vivax* [8]. Nevertheless, other *Anopheles* species might be reintroduced in the future. Thus, it should be highlighted the importance of vector and human cases surveillance for prevention of introduced malaria in non-endemic regions to avoid reemergence situations, as it recently occurred in Greece [7].

## Data Availability

This study involves the use of patient medical data from the Spanish Centralized Hospital Discharge Database (CMBD) (RENAVE). CMBD data are hosted by the Ministry of Health, Consumption and Social Welfare (MSCBS). Researchers working in public and private institutions can request the databases by filling, signing and sending a questionnaire available at the MSCBS website. In this questionnaire a signed Confidentiality Commitment is required. All data are anonymized and de-identified by the MSCBS before it is provided to applicants. According to this Confidentiality Commitment signed with the MSCBS, researchers cannot provide the data to other researchers that must request the data directly to the MSCBS in the following link: https://www.mscbs.gob.es/estadEstudios/estadisticas/estadisticas/estMinisterio/SolicitudCMBDdocs/Formulario_Peticion_Datos_CMBD.pdf. The RENAVE dataset is not publicly available due to restrictions imposed by National Epidemiological Surveillance Network, following a similar policy to other Public health Agencies, as the European Centre for Disease Control. The RENAVE, managed and maintained by the National Centre of Epidemiology, has the mandate to collect, analyse and disseminate surveillance data on infectious diseases in Spain. There is not direct access to the RENAVE database, but requests from third parties not belonging to the RENAVE with research purposes are solved by the National Centre of Epidemiology (http://www.isciii.es/).

## Abbreviations

aOR: adjusted odds ratio
CDC: Centers for Disease Control and Prevention
CMBD: Centralized Hospital Discharge Database
CIBERESP: Consortium for Biomedical Research in Epidemiology and Public Health
ECDC: European Centre for Disease Prevention and Control
EFTA: European Free Trade Association
EU: European Union
IQ: interquartile range
ICD-9CM: International Classification of Diseases, Ninth Revision, Clinical Modification
MSCBS: Ministry of Health, Consumption and Social Welfare
NHS: National Health System
RENAVE: National Network of Epidemiological Surveillance
SiViEs: national reporting electronic platform
RICET: Network Biomedical Research on Tropical Diseases
OR: odds ratio
VFRs: visiting friends and relatives
WHO: World Health Organization
